# 280. Burden of Hyperglycemia in Patients Receiving Dexamethasone for Severe COVID-19

**DOI:** 10.1093/ofid/ofab466.482

**Published:** 2021-12-04

**Authors:** Kirk B Fetters, Stephen Judge, Timothy Hatlen, Eric Daar

**Affiliations:** Harbor-UCLA Medical Center, Torrance, California

## Abstract

**Background:**

Previous studies demonstrated the adverse impact corticosteroids can have on blood glucose homeostasis in both diabetics and non-diabetics. This raises concern for corticosteroid use in severe COVID-19 where the population is enriched for those at highest risk of severe disease, such as diabetics and patients with obesity. Previous studies of dexamethasone in COVID-19 were limited by the inability to assess steroid-induced hyperglycemia or the impact of hyperglycemia on hospital resources.

**Objective:**

The study aimed to describe the clinical characteristics, management, and outcomes related to hyperglycemia, before and after dexamethasone therapy was used as the standard of care in patients with severe COVID-19.

**Methods:**

We performed a pre/post retrospective study of patients with severe COVID-19 pneumonia admitted from May to July 2020 to Harbor-UCLA Medical Center. 126 patients were evaluated. 64 received dexamethasone and 62 did not. To quantify the effect of dexamethasone on diabetic vs. non-diabetic patients, we documented the average blood glucoses and frequency of correctional insulin doses required by each patient group (diabetic with and without dexamethasone, non-diabetic with and without dexamethasone).

**Results:**

While dexamethasone was associated with higher median blood glucose and more frequent correctional insulin dosing in diabetic patients, there was minimal effect of dexamethasone on hyperglycemia in non-diabetic patients. Furthermore, while non-diabetic patients receiving dexamethasone required more doses of correctional insulin per day vs non-diabetic patients not receiving dexamethasone (0.3 doses per day vs 0.1 doses per day), the frequency of correctional insulin doses required by non-diabetics on dexamethasone remained low at 0.3 doses per day.

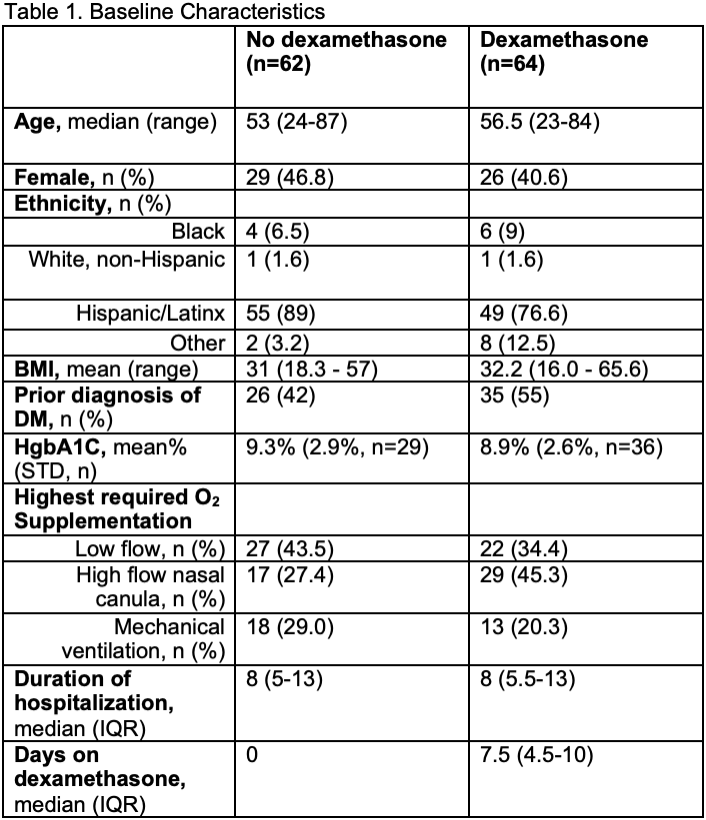

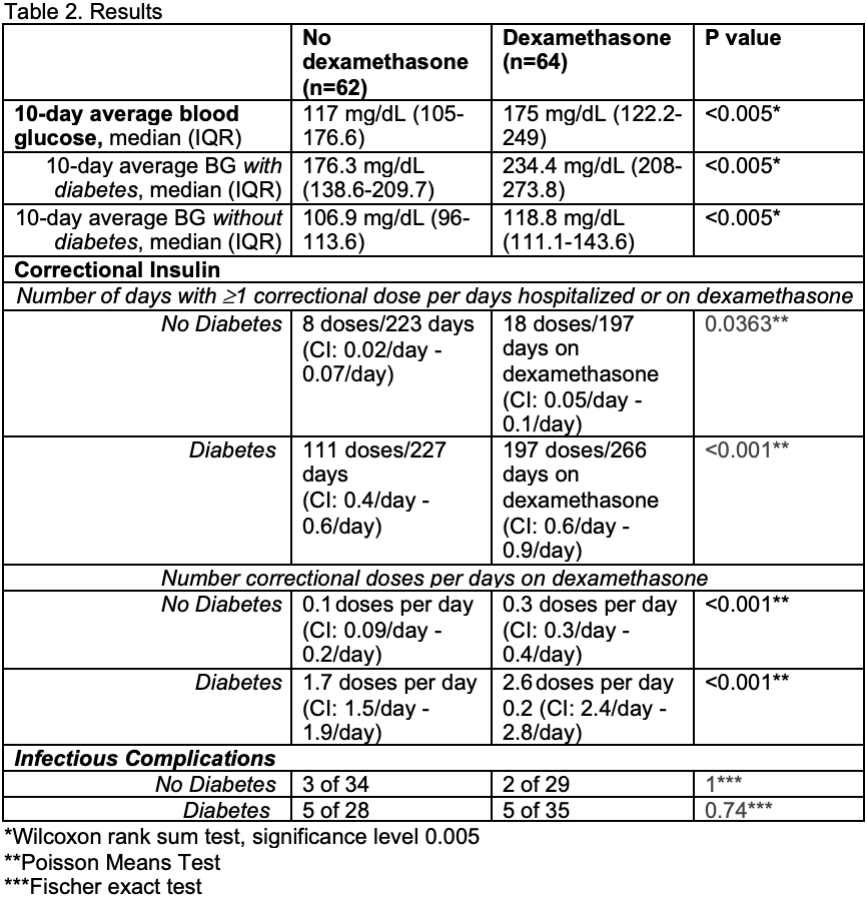

**Conclusion:**

NIH COVID-19 guidelines recommend administering dexamethasone only if the patient is in a monitored setting. Our data support the NIH concerns that outpatients with diabetes receiving steroids are at risk for hyperglycemic complications. However, contrary to the NIH guidelines, our data suggest that patients without diabetes receiving steroids are at low risk for complications due to hyperglycemia and a majority do not require monitoring.

**Disclosures:**

**Eric Daar, MD**, **Gilead** (Consultant)**Gilead** (Research Grant or Support)**Merck** (Research Grant or Support)**Merck** (Consultant)**ViiV** (Research Grant or Support)

